# Exploiting the biological effect exerted by lipid nanocapsules in non-alcoholic fatty liver disease

**DOI:** 10.1016/j.jconrel.2023.03.012

**Published:** 2023-03-15

**Authors:** Inês Domingues, Cecilia Bohns Michalowskia, Valentina Marotti, Wunan Zhang, Matthias Van Hul, Patrice D. Cani, Isabelle A. Leclercq, Ana Beloqui

**Affiliations:** a UCLouvain, Université catholique de Louvain, Louvain Drug Research Institute, Advanced Drug Delivery and Biomaterials Group, Avenue Emmanuel Mounier 73, 1200 Brussels, Belgium; b UCLouvain, Université catholique de Louvain, Louvain Drug Research Institute, Metabolism and Nutrition Group, Avenue Emmanuel Mounier 73, 1200 Brussels, Belgium; c WELBIO (Walloon Excellence in Life sciences and BIOtechnology), WELBIO Department, WEL Research Institute, Avenue Pasteur, 6, 1300 Wavre, Belgium; d UCLouvain, Université catholique de Louvain, Institute of Experimental and Clinical Research, Laboratory of Hepato-Gastroenterology, Avenue Emmanuel Mounier 53, 1200 Brussels, Belgium

**Keywords:** Non-alcoholic fatty liver disease, Non-alcoholic steatohepatitis, Glucagon-like peptide 1, Glucagon-like peptide 1 analog, Lipid Nanocapsules, Oral delivery

## Abstract

Non-alcoholic fatty liver disease (NAFLD) affects approximately 25% of the global adult population and can progress to end-stage liver disease with life-threatening complications; however, no pharmacologic therapy has been approved. Drug delivery systems such as lipid nanocapsules (LNCs) are a very versatile platform, easy to produce, and can induce the secretion of the native glucagon-like peptide 1 (GLP-1) when orally administered. GLP-1 analogs are currently being extensively studied in clinical trials in the context of NAFLD. Our nanosystem provides with increased levels of GLP-1, triggered by the nanocarrier itself, and by the plasmatic absorption of the encapsulated synthetic analog (exenatide). Our goal in this study was to demonstrate a better outcome and a greater impact on the metabolic syndrome and liver disease progression associated with NAFLD with our nanosystem than with the subcutaneous injection of the GLP-1 analog alone. To that end, we studied the effect of chronic administration (one month) of our nanocarriers in two mouse models of early NASH: a genetic model (*foz/foz* mice fed a high fat diet (HFD)) and a dietary model (C57BL/6J mice fed with a western diet plus fructose (WDF)). Our strategy had a positive impact in promoting the normalization of glucose homeostasis and insulin resistance in both models, mitigating the progression of the disease. In the liver, diverging results were observed between the models, with the *foz/foz* mice presenting a better outcome. Although a complete resolution of NASH was not achieved in either model, the oral administration of the nanosystem was more efficient at preventing the progression of the disease into more severe states than the subcutaneous injection. We thus confirmed our hypothesis that the oral administration of our formulation has a stronger effect on alleviating the metabolic syndrome associated with NAFLD than the subcutaneous injection of the peptide.

## 1 Introduction

Non-alcoholic fatty liver disease (NAFLD) is caused by a cluster of intertwined factors (environmental, genetic, lifestyle, etc.) that lead to the slow progression of this highly prevalent metabolic liver disorder [1]. Non-alcoholic fatty liver (NAFL) is considered the first stage and is described as having >5% of hepatocytes containing lipid droplets. Non-alcoholic steatohepatitis (NASH) is a more progressed stage and comprises hepatic steatosis, lobular inflammation, hepatocyte injury with ballooning as a marker and variable degrees of fibrosis. More advanced cases can lead to increased fibrosis, cirrhosis, and hepatocellular carcinoma (HCC). Most patients affected by NAFLD have associated dysmetabolic traits such as obesity and type 2 diabetes mellitus (T2DM), leading to the concept that NAFLD is the hepatic manifestation of the metabolic syndrome (MetS), affecting approximately 25% of the global adult population. Current treatment options are focused on a stage-based approach, with lifestyle modifications (exercise and diet restrictions) being the only validated therapeutic approach. Although this approach has been shown to reverse NASH, it is difficult to maintain in the long run, and currently, there are no approved pharmacological treatments [1,2]. However, there are increasing studies dealing with the potential beneficial effects of e.g., natural products on NAFLD treatment (berberine, curcumin, among others) [3].

Incretin-like hormones such as glucagon-like peptide-1 (GLP-1) act by evoking a glucose-dependent action on insulin secretion, inhibiting glucagon release, slowing down gastric emptying and reducing food intake, leading to weight loss [4]. GLP-1 is rapidly inactivated by the circulating enzyme dipeptidyl peptidase IV (DPP-IV). This prompted the development of GLP-1 analogs with a prolonged half-life, currently approved for the treatment of T2DM and obesity. Patients with NAFLD have reduced GLP-1 levels, and when used as a therapeutic approach, GLP-1 analogs have an indirect impact on hepatic metabolism, reducing hepatic steatosis, inflammation and, to a lesser extent, fibrosis [4,5].

GLP-1 analogs administered as a subcutaneous injection are being extensively studied in clinical trials for the treatment of NAFLD and are a promising therapeutic approach used in mono- or combination therapies. As examples, liraglutide (LEAN Project: ClinicalTrials.gov, number NCT01237119) [6] and semaglutide (ClinicalTrials.gov, number NCT02970942) [7] have both completed phase II clinical trials and met their primary endpoint of NASH resolution without worsening of fibrosis. A phase III clinical trial of semaglutide to treat NASH has already been launched (ClinicalTrials.gov, number NCT04822181).

Our group was the first to demonstrate that oral drug delivery systems such as lipid nanocapsules (LNCs) successfully stimulate the release of endogenous GLP-1 [8,9]. This drug delivery system has the capability of encapsulating, protecting, and facilitating the transport of drugs/peptides through the gastrointestinal tract and into the bloodstream while increasing the physiological levels of GLP-1. Exenatide (EXE), a GLP-1 analog, has been encapsulated into these nanocarriers, and this dual-action strategy has been proven effective in a mouse model of T2DM. This approach has shown effects not only on glucose homeostasis but also on hepatic steatosis, with a better outcome when compared to the exenatide subcutaneous injection [10]. We selected exenatide as a model GLP-1 analog to evaluate this effect in the context of NAFLD treatment. Although GLP-1 analogs with a longer half-life are currently being investigated in clinical trials for the treatment of NAFLD, we hypothesized that increasing endogenous GLP-1 levels would induce sufficient stimulus to treat NAFLD and thus, we could reach therapeutically relevant levels without the need for a long half-life peptide.

The premise of this study is based on the dual-effect provided by exenatide-loaded lipid nanocapsules (EXE-RM-LNCs) when orally administered. We hypothesized that by increasing the secretion of the endogenous GLP-1 and the absorption of a GLP-1 analog by oral administration of this formulation we will have a stronger impact on NAFLD-associated metabolic syndrome than the subcutaneous injection of the GLP-1 analog, preventing the progression into more severe disease states. The present work aimed to evaluate the impact of our nanosystem on the progression of the disease in two different mouse models of early NASH (without fibrosis) when undergoing chronic treatment (one month) versus the subcutaneous injection of the analog alone.

## 2 Materials and methods

### 2.1 Materials

Labrafac® WL 1349 (caprylic/capric acid triglycerides) and Peceol® (oleic acid mono-, di- and triglycerides) were provided by Gattefosśe (Saint-Priest, France). Lipoid® S100 (soybean lecithin at 94% of phosphatidylcholines) was a gift from Lipoid GmbH (Ludwigshafen, Germany). Kolliphor® HS15 (12-hydroxystearate PEG 660 and PEG 660), Span 80® (sorbitan oleate) and sodium chloride (NaCl) were purchased from Sigma-Aldrich (St. Louis, USA). Exenatide was purchased from Bachem (Bubendorf, Switzerland). Dipeptidyl peptidase IV (DPP-IV) inhibitor was purchased from Millipore (St. Charles, USA). All chemical reagents used in this study were of analytical grade.

## 3 Methods

### 3.1.1 Preparation and characterization of the drug delivery system

#### 3.1.1.1 Preparation of reverse micelle-loaded lipid nanocapsules

LNCs were prepared by a phase inversion temperature method using generally recognized as safe (GRAS) materials. Exenatide was encapsulated within reserve micelles prior to its incorporation into LNCs, as previously described by Xu et al. [10] Briefly, exenatide reverse micelles (RMs) were prepared by mixing Labrafac® WL 1349 and Span 80® with high-speed stirring and then adding 50 μL of a 30 mg/mL solution of exenatide in Milli-Q water. LNCs were prepared by weighing and mixing all of the components (Labrafac® WL 1349, Peceol®, Lipoid® S100, Kolliphor® HS15, sodium chloride (NaCl) and Milli-Q water). Then, this mixture was subjected to 3 temperature cycles of heating and cooling (50 °C- 68 °C). During the last cycle, when the temperature was above the phase inversion zone (PIZ; 59 °C -61.5 °C), 500 μL of RM containing exenatide was added to the mixture and allowed to cool down until reaching the PIZ temperature. Finally, 2.5 mL of cold Milli-Q water was added under stirring conditions. Blank LNCs were prepared following the same protocol but without exenatide. The composition of the nanocapsules is summarized in Table I.

#### 3.1.1.2 Characterization of the exenatide-loaded lipid nanocapsules

LNCs were characterized in terms of particle size, polydispersity index (PDI) and zeta potential. The first two parameters were assessed by dynamic light scattering (DLS) and the latter by laser Doppler velocimetry (LSV) using a Zetasizer Nano ZS (Malvern Instruments Ltd., Worcestershire, UK). For the analyses of the zeta potential, 10 μL of LNC was dispersed in 2 mL of Milli-Q water.

The encapsulation efficiency (EE, %) was calculated as follows: the total amount of exenatide was calculated by disrupting the nanoparticles in methanol (50 μL LNC in 950 μL of methanol) followed by strong vortexing. Free exenatide was recovered by ultracentrifugation using Amicon® centrifuge filters (MWCO 100 kDa, 4000 g, 4 °C, 20 min) (Millipore, St. Charles, USA). The resulting filtrates were diluted 2 times. Total and free concentrations of exenatide were quantified using an HPLC method as described below (Section 2.2.1.4). The EE (%) was calculated with the equation:

$$EE\left(\%\right)=\frac{\left(Total\;amount\;of\;exenatide\right)-\left(Free\;exenatide\right)}{\left(Total\;amount\;of\;exenatide\right)}\times 100$$

#### 3.1.1.3 Quantification of exenatide

The encapsulated concentrations of exenatide were quantified by a high-performance liquid chromatography (HPLC, Schimadzu, Japan) gradient method as previously described by Xu et al. [10] A Kinetex® EVO C18 column (100 Å, 2.6 μm, 150 × 4.6 mm) with a security guard column was used (Phenomenex, USA). The aqueous and organic mobile phases consisted of 0.05% (v/v) of trifluoroacetic acid (TFA) in water and acetonitrile, respectively. This approach uses a gradient method with an initial ratio of 10:90 (v/v, aqueous: organic phase) at a flow rate of 1 mL/min linearly changing to 90:10 over 10 min and kept constant for one minute. After that, the ratio linearly changes to the initial composition during the next 1.5 min and stabilizes for one minute. The volume injected was 20 μL and the detection wavelength was 220 nm with a retention time of 6.9 min. The limits of detection and quantification were 1.1 ± 0.4 μg/mL and 3.3 ± 1.1 μg/mL, respectively [10].

### 3.1.2 In vivo studies

#### 3.1.2.1 Animals

All animal studies were approved by and performed in accordance with the local animal committee under references 2021/ UCL/MD/055 and 2020/UCL/MD/018.

#### 3.1.2.2 Total GLP-1 secretion in a mouse model of NAFLD

*Foz/foz* mice were randomly divided into two groups after 9 weeks of highfat diet (HFD) intake, with 6 mice per group. The animals were fasted for 4 h and 1 h prior to the glucose challenge (40 mg of glucose per mouse) the blank nanoparticles (1.62 mg/g) were orally administered to the RM-LNC group, while the control (CTRL) group received an equivalent volume of Milli-Q water by gavage. Blood was collected from the tail vein in the presence of DPP-IV inhibitor (20 μL per mL of blood), 30 min after the oral administration and 15 min after the glucose challenge. The blood samples were centrifuged (3000 *g*, 15 min at 4 °C) and the plasma collected was stored at −80 °C until further analysis. Total GLP-1 levels and insulin levels were quantified using ELISA kits (ELISA kit, K1503PD Meso Scale Discovery, USA; Ultrasensitive Insulin ELISA kit, 10–1132-01 Mercodia, Uppsala, Sweden).

#### 3.1.2.3 Oral glucose tolerance test (OGTT)

The mice were fasted for 4 h, after which a glucose challenge (40 mg of glucose per mouse) was given by gavage. Glucose values were measured before (−30 and 0 min) and after glucose administration at 15, 30, 60, 90, 120, and 180 min with a glucometer (Glucometer Accu-Check Aviva). Blood from the tail vein (~60 μL) was also collected before and after (−30 and 15 min) the glucose challenge using a heparin (3.0 IU/capillary) coated capillary (Hirschmann 9,100,260). The plasma was then retrieved after blood centrifugation (3000 *g*, 15 min at 4 °C) and stored at −80 °C until insulin assessment (Ultrasensitive Insulin ELISA kit, 10–1132-01 Mercodia, Uppsala, Sweden).

#### 3.1.2.4 Long-term treatment studies in two animal models of early NASH

Four-week-old male *foz/foz* (*Alms1*^–/–^) mice on a NOD⋅B10 background were kept on a high fat diet, 60 kcal% fat (HFD, D12492 ResearchDiets, USA) for 12 weeks (*n* = 4–6/group) [11]. During the 11th week of disease induction, an OGTT was performed according to the method described above (Section 2.2.2.3).

Eight-week-old male C57BL/6J mice were kept on a normal diet (ND, SAFE Diets A03) or on a western diet, 0.5% cholesterol (D05011404 ResearchDiets, USA) plus 30% (*w*/*v*) fructose (F0127, Sigma-Aldrich) in the drinking water for 20 weeks (WDF) (*n* = 10/group) [12]. Their weight was monitored weekly. During the 18th week of disease induction and during the last week of treatment, the mice underwent a 4 h fasting period (the food was removed, and the fructose water was replaced by normal water), after which fasting glycemia was measured and blood from the tail vein was collected (~60 μL) for insulin assessment.

The mice underwent treatment for 1 month while continuing the diet intake. Daily administrations, by gavage or subcutaneous injection, were given at the same time every day (3:30 pm), their weight was monitored, and non-fasting glycemia was measured every week. Exenatide was administered orally in solution (EXE) or encapsulated within reverse micelle lipid nanocapsules (EXE-RM-LNCs) (500 μg/kg), or subcutaneously in solution (EXE SC) (10 μg/kg). One mouse group received the corresponding concentration of unloaded lipid nano-capsules (RM-LNCs) orally. Control groups (CTRL HFD/CTRL ND/CTRL WDF) were given an equivalent volume of Milli-Q water by gavage. The volumes administered were based on the total amount of exenatide present in the formulation using the HPLC method described above (Section 2.2.1.3).

Regarding the doses used within this study, we are administering a 50-times lower dose subcutaneously compared to the oral dose. This is based on previous pharmacokinetic studies conducted by our group in both normoglycemic mice and type 2 diabetic mice that showed a relative bioavailability of EXE (when encapsulated within LNCs) of ~4% in diabetic mice [10].

After the treatment period, the mice were anesthetized with isoflurane (Isoflutek®, Karizoo Laboratories), and blood from the portal and cava vein was retrieved in the presence of a DPP-IV inhibitor (20 μL per mL of blood), after which the mice were euthanized by cervical dislocation. The blood collected was centrifuged (3000 *g*, 15 min at 4 °C), and the plasma was stored at −80 °C until further analyses. Active GLP-1 was measured in portal plasma (ELISA kit, K1503OD Meso Scale Discovery, USA), whereas total GLP-1 (ELISA kit, K1503PD Meso Scale Discovery, USA) and liver enzymes (AST/ALT) (DRY-CHEM NX500, Fujifilm) were measured in systemic plasma.

At sacrifice, the liver was collected and weighed. A piece of the liver was immersed in 4% paraformaldehyde (PFA) and embedded in paraffin for histological analysis. Another piece was immediately snap frozen in lipid nitrogen and stored at −80 °C. The total lipid content was measured by extracting the lipids from frozen livers with methanol and chloroform and quantifying with the vanillin phosphoric acid reaction [13].

#### 3.1.2.5 Histology and Immunohistochemistry

The fixed liver sections were stained with hematoxylin and eosin (H&E) or used for immunohistochemical detection of macrophages and neutrophils. Briefly, a polyclonal rat anti-mouse F4/80 antibody (1:200, AbD Serotec MCA497G), LY-6G antibody (1:2000, BD Pharmingen 551,459), polyclonal rabbit anti-rat antibody (1:100, Vector AI-4001) and an envision anti-rabbit HRP antibody (Dako K4003) were used, followed by a diaminobenzidine (Dako K3468)) to reveal the peroxidase activity and the sections were counterstained with hematoxylin. Macrophages and neutrophils were quantified as the F4/80+ and LY6G+ areas (% total area), respectively, by using the QuPath software [14]. The NAFLD Activity Score (NAS) was assessed as previously described by Kleiner et al. [15] Briefly, NAS is defined as the sum of 3 histological features of NAFLD: steatosis ranging from 0 to 3 (0- < 5%; 3- > 66%), ballooning ranging from 0 to 2 (0-none; 2-many) and finally lobular inflammation ranging from 0 to 3 (0-no foci; 3- > 4 foci). Hepatic crown-like structures (hCLS), which are represented by macrophages surrounding dead or dying hepatocytes with large lipid droplets, were quantified in 10 high-magnification (x40) fields per mouse

### 3.2 Statistical analysis

The GraphPad Prism 9 (California, USA) program was used to perform all statistical analyses. Data are presented as the mean ± standard error of the mean (SEM) with outliers removed based on the Grubb’s test. Normality was assessed by using the Shapiro-Wilk test, and for comparisons involving multiple groups, a two-way or one-way ANOVA was performed followed by Tukey’s multiple comparisons test. A Kruskal-Wallis’s test was used followed by Dunn’s multiple comparisons test for non-parametric analysis of multiple groups. For comparisons between 2 groups, an unpaired *t*-test or Mann-Whitney test was used to assess significant differences. A difference of *P* < 0.05 was considered statistically significant.

## 4 Results & discussion

### 4.1 LNC characterization

**Fig. 1:**
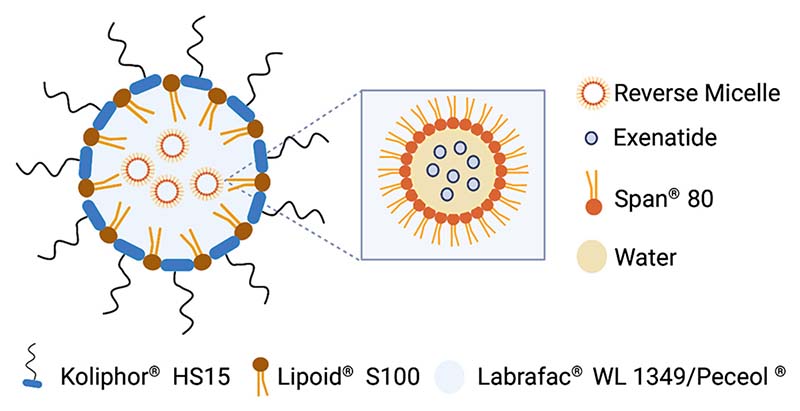
Schematic representation of the exenatide-loaded lipid nanocapsules.

LNCs with and without EXE were prepared using generally recognized as safe (GRAS) excipients, and their composition is described in Table 1. A phase inversion method was performed as previously described [10]. A schematic representation of the nanosystem is shown in Fig. 1. The average size obtained was ∼180 nm, with a homogeneous population of nanoparticles (PDI *<* 0.2) and a negative surface charge (−7 mV). The encapsulation efficiency was concordant with previous results (~80%) (Table 2).

### 4.2 RM-LNC effect is preserved in a mouse model of NAFLD

Our group was the first to demonstrate that lipid nanocapsules could, when orally administered, stimulate the release of the native GLP-1 in a mouse model of T2DM [10]. To demonstrate that the effect observed previously was maintained in NAFLD, we used a genetic mouse model of the disease, the *foz/foz* model (Section 3.3). We orally administered RMLNCs and measured total GLP-1 plasma levels. The results obtained were concordant with our previous observations with a ~ 3-fold increase in plasmatic levels (*P* = 0.0022) 30 min after oral administration and ã 2-fold increase (*P* = 0.0076) in the plasmatic values measured 15 min after the glucose challenge, when compared to the CTRL group (Supplementary information, Fig. S1). Based on these results, we confirmed that the effect exerted by RM-LNCs on GLP-1 secretion was preserved in this disease model.

### 4.3 NAFLD is successfully induced in two different murine models

We chose 2 different mouse models of NAFLD presenting symptoms of an important metabolic syndrome: a genetic model (*foz/foz* – *Alms1*^–/–^) and a dietary model (WDF). *Foz/foz* mice lack the *Alms1* gene, which induces a generalized ciliopathy leading to several important metabolic alterations, including hyperphagia, which contributes to the development of obesity and severe insulin resistance in mice. A HFD intake accelerates disease development into NASH, which can progress to liver fibrosis [16,17]. The western diet-induced mouse model is characterized by high fat, high cholesterol, and high fructose intake mimicking the *“fast-food diet”* and leading to the development of the metabolic syndrome [12]. In the *foz/foz* model, the diet used was a high-fat diet with 60% Kcal of fat, while the C57BL/6 J mice were fed a western diet with 0.5% cholesterol, 40% Kcal of fat and 30% (*w*/*v*) fructose in their drinking water.

For this study, we aimed to evaluate the efficacy of our treatment at preventing the progression of the disease. The disease induction periods chosen within these studies were selected based on the characteristics of early NASH (hepatic steatosis, inflammation, and hepatocyte injury), without the presence of fibrosis. We confirmed that the phenotype between the two models was different regarding body weight, glucose levels, and insulin profiles.

*Foz/foz* mice were obese with an average weight of 74.9 ± 1.5 g, presented high glucose levels with an average fasting glycemia of 413.6 ± 28.2 mg/dL, and were severely insulin resistant after 12 weeks of HFD intake with an average plasma insulin of 38.3 ± 7.8 ng/mL and a homeostatic model assessment of insulin resistance (HOMA-IR) of 31.2 ± 5.0 (Supplementary information, Fig. S2).

C57BL/6 J mice fed a WDF were obese with an average weight of 48.1 ± 0.6 g, and their overall glucose levels (x^−^=146.3 ± 3.2 mg/dL) were maintained with the presence of hyperinsulinemia (x^−^=2.7 ± 0.2 ng/mL) after 20 weeks of diet intake with an average HOMA-IR of 1.0 ± 0.1. In contrast, the C57BL/6 J mice fed a ND were on average 34.1 ± 0.9 g in weight, and although they exhibited glucose values similar to those of the mice fed a WDF diet (x^−^=148.6 ± 5.2 mg/dL), their insulin values were in the normal range (x^−^=1.1 ± 0.2 ng/mL) with low HOMAIR results (x^−^=0.4 ± 0.1) (Supplementary information, Fig. S3). The mice from both models (*foz/foz* and WDF) were randomized into the different treatment groups in order to obtain body weight-matched groups (Supplementary information, Fig. S2 and S4).

When comparing both models, the differences in glucose metabolism were marked, with the *foz/foz* mice showing characteristics of profound insulin resistance and the C57BL/6J mice presenting a pre-diabetic profile with glucose tolerance preserved by compensatory increased circulating levels of insulin.

### 4.4 Effect of exenatide-loaded lipid nanocapsules on glucose homeostasis and insulin resistance in two animal models of early NASH

#### 4.4.1 EXE-RM-LNCs have a positive impact on glucose homeostasis and insulin resistance in the foz/foz early NASH model

**Fig. 2:**
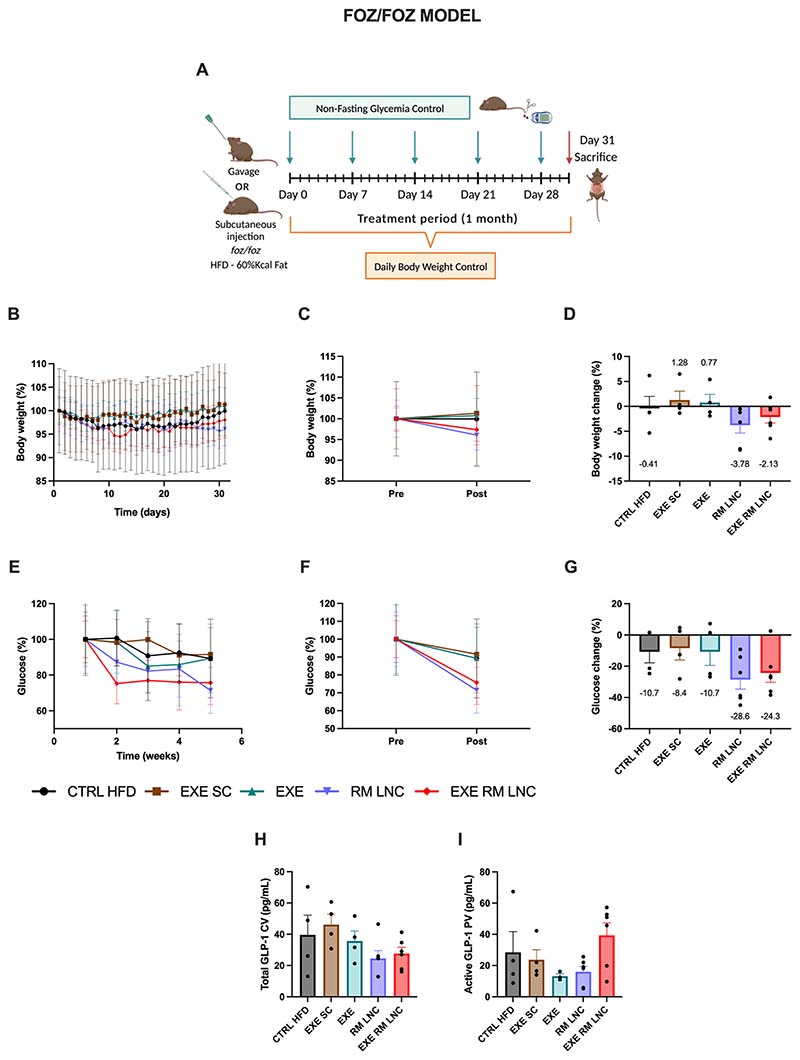
EXE-RM-LNCs have an impact on glucose homeostasis and insulin resistance in the *foz/foz* early NASH model. (A) Schematic representation of the treatment period, (B) Body weight (%), (C) Pre/Post: Body weight (%), (D) Body weight change (%), (E) Weekly non-fasting glucose (%), (F) Pre/Post: Non-fasting glucose (%), (G) Non-fasting glucose change (%), (H) Total GLP-1 levels (pg/mL) measured in systemic plasma, (I) Active GLP-1 levels (pg/mL) measured in portal plasma. Pre: beginning of treatment; Post: end of treatment. The results shown in (D; G) were calculated by subtracting the post values from the pre values. Data are represented as the mean ± SEM (*n* = 3–6). CV: Cava vein; PV: Portal vein.

The current therapeutic approach to NAFLD treatment is based on lifestyle modifications. A weight loss of 5 to 10% has been shown to be of great importance in the resolution of the disease [1]. Throughout the 1-month treatment, only the RM-LNC and EXE-RM-LNC groups decreased their body weights by 3.8 ± 1.6% and 2.1 ± 1.2%, respectively (Fig. 2B-D) (Supplementary information, Fig. S5A–C).

Non-fasting plasma glucose values were gradually reduced throughout the treatment period in both the RM-LNC-treated and EXE-RM-LNC-treated mice (Fig. 2E). When comparing the initial with the final glycemia levels, we can see a marked drop with both unloaded and loaded nanoparticles reducing glycemia by approximately 25 to 30% (RM-LNC: x^−^= −28.6 ± 6.1% and EXE-RM-LNC: x^−^= −24.3 ± 6.0%) while the CTRL HFD and EXE SC groups had an average decrease of 10.7 ± 7.1% and 8.4 ± 7.6% decrease, respectively (Fig. 2F-G). Taking into consideration the severity of hyperglycemia in these mice at the beginning of the treatment with average starting values of glucose for both the EXE-RM-LNC and RM-LNC groups of nearly 400 mg/dL (EXE-RM-LNC: x^−^=432.7 ± 44.5 mg/dL vs. x^−^=327.5 ± 52.8 mg/dL and RM-LNC: x^−^=445.3 ± 68.1 mg/dL vs. x^−^=318.2 ± 57.3 mg/dL), it is noteworthy that our nanosystem exerted such an impact on glucose reduction (Supplementary information, Fig. S5D–F).

Despite the decrease in the glucose levels, circulating glucose values were not normalized by the end of the treatment. We would like to highlight that the mice underwent only 1month of treatment. Considering the 48–72 weeks of treatment in ongoing clinical trials with GLP-1 analogs [6,7], one might hypothesize that a prolonged treatment period, provided that a downward trend is maintained, or higher exenatide doses, might could possibly help attain normal glycemia levels.

Both unloaded and loaded formulations exhibited similar results in terms of non-fasting glycemia, which could be explained by the increased levels of endogenous GLP-1 provided by the nanoparticles themselves. We previously demonstrated this secretory effect [10] in a mouse model of type 2 diabetes, and we have confirmed it in the NAFLD disease model (Supplementary information, Fig. S1). These GLP-1 levels could result in a greater impact on the metabolic syndrome associated with NAFLD, thus outperforming the effect of the subcutaneous injection.

Total and active GLP-1 levels were measured in the plasma retrieved from both the cava and portal vein at the time of the sacrifice, representing the levels throughout the 1-month treatment. It should be noted that the formulations were not administered on this day, and thus, these GLP-1 levels do not correspond to those induced by the nanoparticles. The mice were also not fasted before blood collection, and since GLP-1 secretion is enhanced by a factor of 2 to 3 after food intake [18], its plasmatic concentration could have been influenced by the amount of food ingested by the mice prior to the sacrifice. However, we can see that with the EXE-RM-LNC-treated group, the active GLP-1 measured in portal plasma is higher than in the other groups (x^−^=39.4 ± 8.0 pg/mL) (Fig. 2I), although not significantly different. The total GLP-1 values were also measured in systemic plasma, and no significant differences were observed among the groups (Fig. 2H).

#### 4.4.2 EXE-RM-LNC impact on glucose homeostasis and insulin resistance is preserved between mouse models of early NASH

**Fig. 3:**
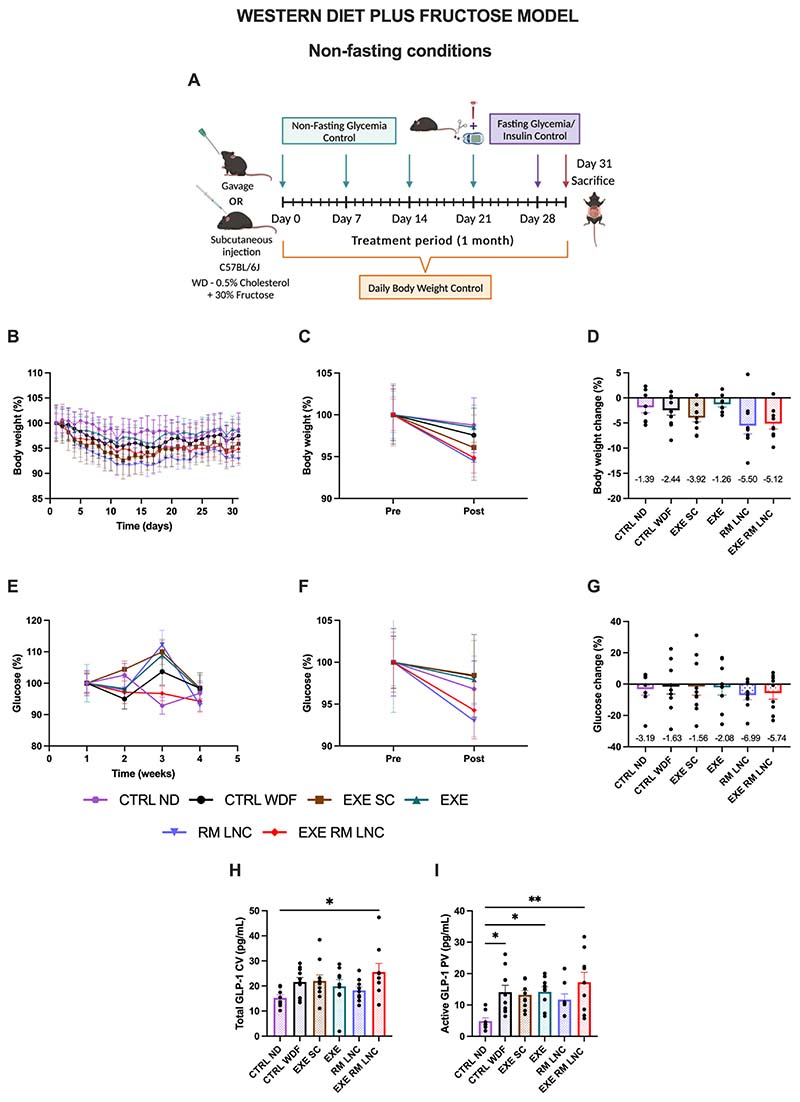
EXE-RM-LNCs have an impact on glucose homeostasis and insulin resistance in the WDF model of early NASH. (A) Schematic representation of the treatment period (1 month), (B) Body weight (%), (C) Pre/Post: Body weight (%), (D) Body weight change (%), (E) Weekly non-fasting glucose (%), (F) Pre/Post: Non-fasting glucose (%), (G) Non-fasting glucose change (%), (H) Total GLP-1 levels (pg/mL) measured in systemic plasma, (I) Active GLP-1 levels (pg/mL) measured in portal plasma. Pre: beginning of treatment; Post: end of treatment. The results presented in (D; G) results were calculated by subtracting the post values from the pre values. Data are represented as the mean ± SEM (*n* = 8–10). *P* values in (H–I) were determined by ordinary one-way ANOVA followed by Tukey’s post hoc test (*P < 0.05; **P < 0.01). CV: Cava vein; PV: Portal vein.

WDF mice exhibited a less severe phenotype with less excess weight and lower starting glucose levels than *foz/foz* mice (Supplementary information, Figs. S2/S4). Nevertheless, the treatment with RM-LNCs as well as with EXE-RM-LNCs achieved a more pronounced decrease in body weight in WDFs than in the *foz/foz* mice, showing weight losses of 5.5 ± 1.6% and 5.1 ± 1.0%, respectively (Fig. 3B-D) (Supplementary information, Fig. S6A–C). This difference in body weight reduction between the 2 models might be due to *foz/foz* mice being hyperphagic, mitigating this way one of the therapeutic effects of the endogenous GLP-1/EXE which slows gastric emptying, inducing satiety and ultimately leading to weight loss [4,5].

When comparing non-fasting glucose values, the effect of both unloaded and loaded formulations was less marked in this model, probably due to the lower starting values of glucose (x^−^=146.3 ± 3.2 mg/dL vs. x^−^=413.6 ± 28.2 mg/dL) (Supplementary information, Figs. S2 and S3). During the 1st week of treatment, EXE-RM-LNC-treated mice had an average glycemia of 151.1 ± 4.7 mg/dL and it was 142.4 ± 5.2 mg/dL one week prior to the sacrifice. Regardless of this, we observed a reduction in glucose levels in the RM-LNC and EXE-RM-LNC groups of 6.9 ± 2.9% and 5.7 ± 3.9%, respectively, and only a decrease of approximately 1% for both the CTRL WDF (x^−^= −1.6 ± 4.7%) and EXE SC (x^−^= −1.6 ± 5.5%) groups (Fig. 3E-G) (Supplementary information, Fig. S6D–F). This shows that the effect of our nanosystem is not model specific, and follows the same profile, although to a lesser extent, in the second model.

Similar to the study conducted with the *foz/foz* mice, total and active GLP-1 levels were measured under the same conditions, meaning that the formulations were not administered on the day of the sacrifice, and the mice were not fasted. Similar results were obtained with increased levels of active GLP-1 in the EXE-RM-LNC-treated mice (x^−^=17.3 ± 3.2 pg/mL). Total GLP-1 values were also quantified, with the only significant differences between the healthy control and the EXE-RM-LNC group (*P* = 0.0398) (Fig. 3H-I). The presence of fructose in the drinking water leads to increased levels of GLP-1 secretion in mice [19], which could explain the higher values of active GLP-1 in the groups fed with a WDF and lower levels for the ND-no-fructose fed mice.

**Fig. 4:**
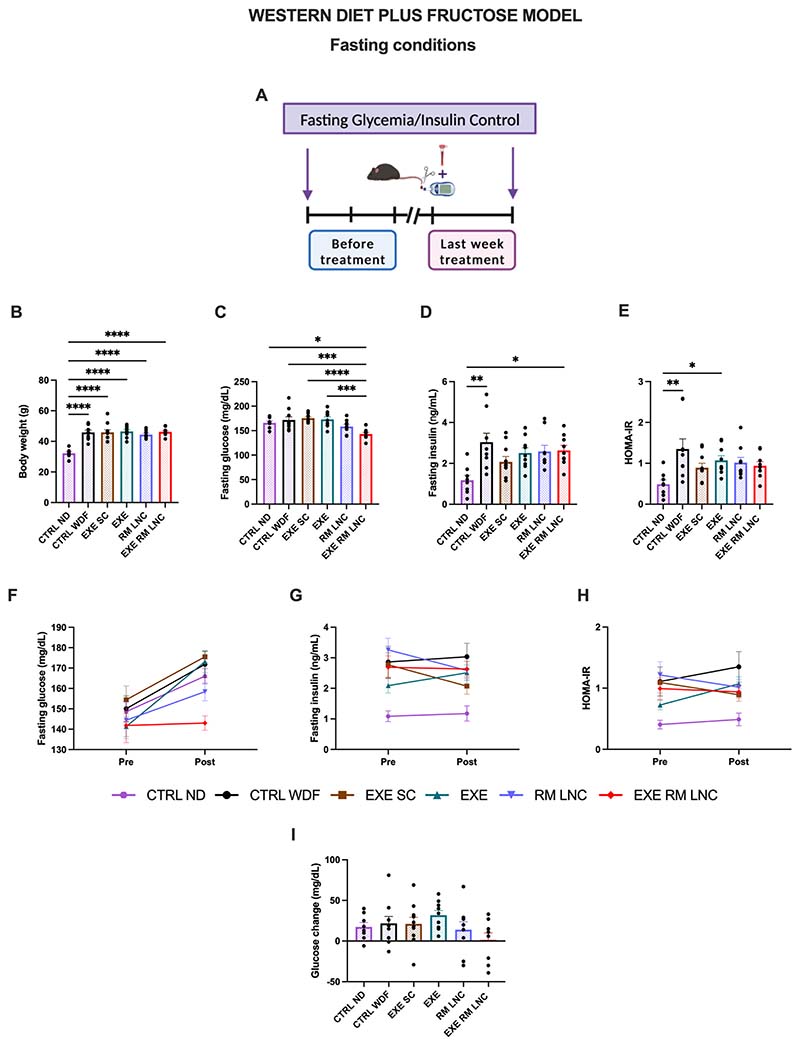
EXE-RM-LNCs impact on glucose homeostasis and insulin resistance under fasting conditions before and at the end of treatment in the WDF-induced model of early NASH. (A) Schematic representation of fasting glycemia and insulin control measurements, (B) Body weight (g), (C) Fasting glucose (mg/dL), (D) Fasting insulin (ng/mL), (E) HOMA-IR, (F) Pre/Post: Fasting glucose (mg/dL), (G) Pre/Post: Fasting insulin (ng/mL), (H) Pre/Post: HOMA-IR, (I) Fasting glucose change (%). Pre: before treatment; Post: last week of treatment. The results (B-E) were obtained in the last week of the treatment period. The results (F–H) compare the results obtained before and at the end of the treatment period. The results shown in (I) were calculated by subtracting the post from the pre values. Data are represented as the mean ± SEM (*n* = 8–10). *P* values in (B-E) were determined by ordinary one-way ANOVA followed by Tukey’s post hoc test or by Kruskal-Wallis followed by Dunn’s post hoc test (*P < 0.05; **P < 0.01; ***P < 0.001; ****P < 0.0001).

Fasting glycemia was recorded during the 18th week of disease induction and at the end of the treatment (Fig. 4A). In the last week, the EXE-RM-LNC group proved to be significantly different from all groups, except with the RM-LNC-treated mice, with almost normalized glycemia (x^−^=143.0 ± 3.5 mg/dL) (Fig. 4C). However, all the groups showed high plasmatic insulin levels (>2 ng/mL) (Fig. 4D). When comparing the results obtained before the beginning of the treatment (Supplementary information, Fig. S3) to those at the end, we can see that the EXE-RM-LNC group had an intermediary phenotype with no significant decrease or increase in any of the parameters analyzed (fasting glucose, insulin, and HOMA-IR), which demonstrates that EXE-RM-LNCs were able to mitigate the progression of insulin resistance (Fig. 4F-I). As previously hypothesized, extending the treatment period or increasing the dose might lead to greater improvement in the overall metabolic syndrome. It should also be considered that as a proof-of-concept, we encapsulated a GLP-1 analog with a short half-life (exenatide, ~2.5 h) and that current clinical studies are evaluating compounds with much longer half-lives, such as 13 h (liraglutide) and 165 h (semaglutide). Replacing exenatide with one of these peptides might also lead to improved outcomes of the treatment.

RM-LNCs per se lowered glucose levels. This was observed in both the *foz/foz* and C57BL/6J mice, with glucose reductions comparable between the two formulation-treated groups (*foz/foz* model with x^−^= −127.2 ± 27.1 mg/dL for RM-LNCs and x^−^= −105.2 ± 26.0 mg/dL for EXE-RM-LNCs vs. WDF model with x^−^= −11.1 ± 4.7 mg/dL for RMLNCs and x^−^= −8.7 ± 5.9 mg/dL for EXE-RM-LNCs) (Supplementary information, Fig. S4F and S5F). With regard to the results observed under fasting conditions with the WDF model, although an increase in glycemia could be observed with the RM-LNC group when compared with the results prior to the treatment (Pre: x^−^=144.4 ± 9.1 mg/dL vs. Post: x^−^=158.4 ± 4.5 mg/dL), a decrease was seen concerning fasting insulin values (Pre: x^−^=3.3 ± 0.4 ng/mL vs. Post: x^−^=2.6 ± 0.3 ng/mL), leading to lower values of the HOMA-IR score (Pre: x^−^=1.2 ± 0.2 vs. Post: x^−^=1.0 ± 0.1) (Fig. 4F-I). We believe that the change in GLP-1 levels triggered post-administration could explain the pronounced effect exerted by the nanoparticles alone.

### 4.5 Effect of exenatide-loaded lipid nanocapsules on liver steatosis and inflammation

#### 4.5.1 EXE-RM-LNCs have an impact on liver steatosis and inflammation in the foz/foz early NASH model

**Fig. 5:**
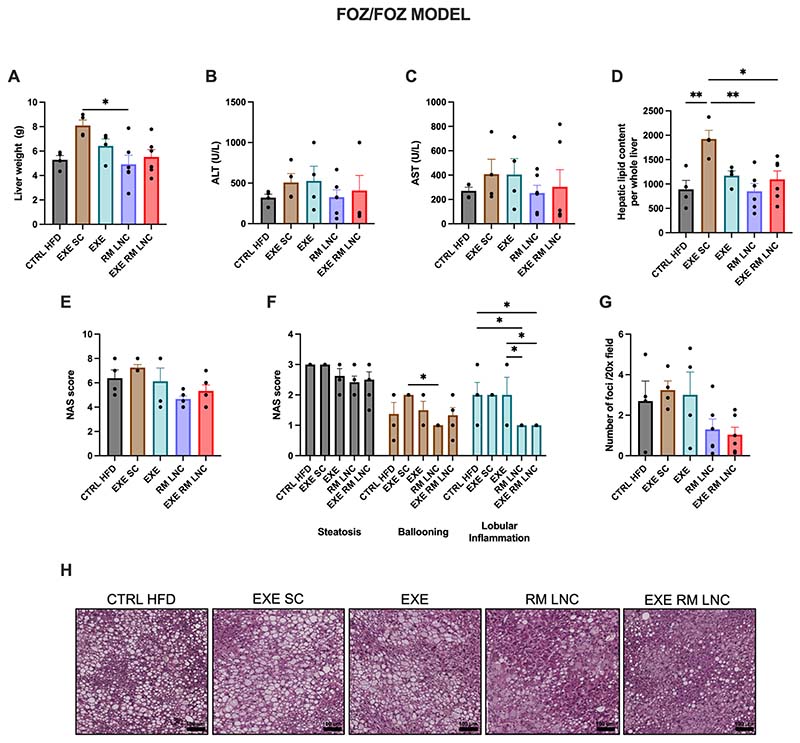
EXE-RM-LNCs have an impact on liver steatosis and inflammation in the *foz/foz* early NASH model. (A) Liver weight (g), (B) ALT levels (U/L) measured in systemic plasma, (C) AST levels (U/L) measured in systemic plasma, (D) Hepatic lipid content per whole liver, (E) Histological NAFLD activity score (NAS), (F) Steatosis, ballooning, and lobular inflammation individual scores, (G) Inflammatory foci per 20× field, (H) Representative H&E liver sections (scale bar: 100 μm). Data are represented as the mean ± SEM (*n* = 4–6). *P* values in (A; D) were determined by ordinary one-way ANOVA followed by Tukey’s post hoc test. *P* values in (F) were determined by a two-way ANOVA followed by Tukey’s post hoc test (*P < 0.05; **P < 0.01).

We found that the liver weights of the RM-LNC (x^−^=4.9 ± 0.7 g) and EXE-RM-LNC-treated mice (x^−^=5.5 ± 0.6 g) were not significantly different compared to that of the CTRL HFD (x^−^=5.2 ± 0.3 g), but that of the RM-LNC group was significantly lower when compared to the EXE SC group (x^−^=8.1 ± 0.5 g). We found the liver weight of EXE-RM-LNC mice to be different when compared to the EXE SC group by an un-paired *t*-test (*P* = 0.015 for EXE LNC (x^−^=5.5 ± 0.6 g) vs. EXE SC (x^−^=8.1 ± 0.5 g)) (Fig. 5A). However, the statistical significance was lost when analyzed by ordinary one-way ANOVA followed by Tukey’s post hoc test. Regarding plasmatic transaminase levels (ALT/AST), there were no statistically significant differences observed for both enzymes, but the RM-LNC and EXE-RM-LNC-treated mice exhibited a strong tendency toward normalized values of both circulating enzymes (Fig. 5B-C). The hepatic total lipid content was quantified and differences between the EXE-RM-LNC, LNC and CTRL HFD groups were encountered when compared with the EXE SC group (Fig. 5D).

According to the NAFLD activity score (NAS), when analyzed by Mann-Whitney test, the RM-LNC group was different when compared to the CTRL HFD and the EXE SC groups (*P* = 0.048 LNC vs. CTRL HFD; *P* = 0.005 LNC vs. EXE SC) and the EXE-RM-LNC group was also different when compared to the EXE SC group (*P* = 0.029 EXE LNC vs. EXE SC) (Fig. 5E). However, the significant difference was lost when analyzed by Kruskal-Wallis followed by Dunn’s post hoc test. The NAS score is the sum of 3 features of NAFLD: steatosis, ballooning, and lobular inflammation [15]. When the scores for each distinct feature were analyzed separately, we observed differences in 2 of the subcategories (ballooning and inflammation). Regarding hepatocyte injury (ballooning) assessed by the visualization of H&E liver slides, a significant difference was observed between the RM-LNC and EXE SC groups (*P* = 0.046 for RM-LNC vs. EXE SC). Interestingly, both loaded and unloaded LNCs had an impact on liver inflammation that was significantly different from the CTRL HFD and EXE group scores (*P* = 0.046 for CTRL HFD vs. RM-LNC and *P* = 0.046 for EXE-RM-LNC and EXE vs. RM-LNC and EXE-RM-LNC) (Fig. 5F). To assess any further impact on inflammation, the number of inflammatory infiltrates was counted in high-magnification fields using H&E liver slides with both formulation groups having a reduced foci count with an average of 1 foci per field (RM-LNC: x^−^=1.3 ± 0.5 foci and EXE-RM-LNC: x^−^=1.0 ± 0.4 foci) as compared to more than double in the other groups (CTRL HFD: x^−^=2.7 ± 1.0 foci, EXE SC: x^−^=3.2 ± 0.5 foci and EXE: x^−^=3.0 ± 1.1 foci). When analyzed by unpaired *t*-test there were significant differences between both formulation groups when compared to the EXE SC group (*P* = 0.0055 for EXE-RM-LNC vs EXE SC; *P* = 0.029 for RM-LNC vs EXE SC). However, when analyzed by ordinary one-way ANOVA followed by Tukey’s post hoc test, the significant difference was lost (Fig. 5G). Histological analyses by H&E revealed a decrease in hepatic steatosis in both RM-LNC-treated and EXE-RM-LNC-treated mice compared with EXE SC-treated and CTRL HFD mice (Fig. 5H). Further analyses were conducted to assess macrophage infiltration by performing a F4/80 immunostaining (F4/80+ area per total area stained) and by counting hepatic crown-like structures (hCLS), which correlate positively with disease severity [20] and are represented by dead or dying hepatocytes surrounded by macrophages, with no significant differences present for both analyses (Supplementary information, Fig. S6A–C).

The effect that we observed in the liver with our treatment is likely through the indirect actions of GLP-1 since there are no GLP-1 receptors (GLP-1R) expressed on hepatocytes, hepatic stellate cells or Kupffer cells [5]. Nonetheless, through the direct action of GLP-1 on insulin and glucagon secretion and through the interaction with GLP-1R+ cells in the central nervous system contributing to weight loss, GLP-1 and GLP-1 analogs can impact hepatic steatosis and inflammation. However, the effects observed beyond weight loss are still not fully understood [5].

#### 4.5.2 EXE-RM-LNCs impact on liver steatosis and inflammation is not preserved between mouse models of early NASH

**Fig. 6:**
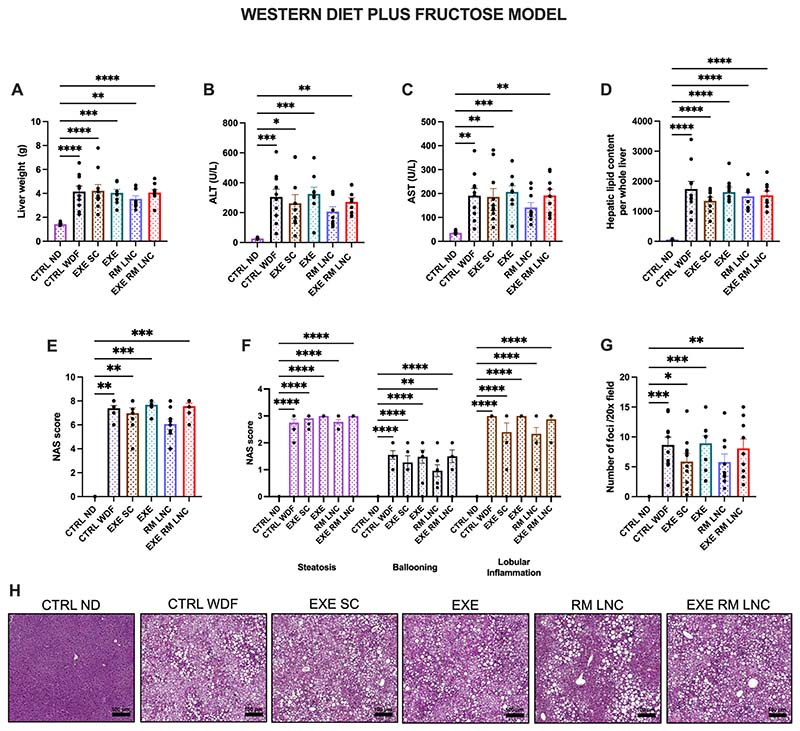
EXE-RM-LNCs impact on liver steatosis and inflammation in the WDF model of early NASH. (A) Liver weight (g), (B) ALT levels (U/L) measured in systemic plasma, (C) AST levels (U/L) measured in systemic plasma, (D) Total lipid content per whole liver, (E) Histological NAFLD activity score (NAS), (F) Steatosis, ballooning, and lobular inflammation individual scores, (G) Inflammatory foci per 20× field, (H) Representative H&E liver sections (scale bar: 100 μm). Data are represented as the mean ± SEM (*n* = 8–10). *P* values in (A-E; G) were determined by ordinary one-way ANOVA followed by Tukey’s post hoc test or by Kruskal-Wallis followed by Dunn’s post hoc test. *P* values in (F) were determined by two-way ANOVA followed by Tukey’s post hoc test (*P < 0.05; **P < 0.01; ***P < 0.001; ****P < 0.0001).

The C57BL/6 J mice had slightly lighter livers than the *foz/foz* mice, and there were no significant differences between the untreated and treated mice fed a WDF (CTRL WDF: x^−^=4.2 ± 0.4 g; EXE SC: x^−^=4.2 ± 0.5 g; EXE: x^−^=4.1 ± 0.3 g; RM-LNC: x^−^=3.5 ± 0.3 g and EXE-RM-LNC: 4.1 ± 0.2 g). The only difference observed, as expected, was with the untreated mice fed a ND (CTRL ND: x^−^=1.4 ± 0.05 g) (Fig. 6A). The same could be observed for the hepatic total lipid content (Fig. 6C). Regarding plasma transaminase levels (ALT/AST), we can see comparable values between the EXE-RM-LNC and EXE SC-treated mice with the RM-LNC group (ALT: x^−^=207.0 ± 32.6 U/L; AST: x^−^=141.8 ± 20.5 U/L), and this was the only measure not significantly different from the ND-fed mice (Fig. 6B-C).

Regarding the NAS score, the RM-LNC group was the only group not significantly different from the healthy control group, and when analyzed by the Mann-Whitney test, the RM-LNC group was significantly different from the CTRL WD, EXE and EXE-RM-LNC groups (*P* = 0.0152 RM-LNC vs. CTRL WDF; *P* = 0.005 RM-LNC vs. EXE; *P* = 0.0103 RM-LNCs vs. EXE-RM-LNCs) (Fig. 6E). In this model, when the different features of the NAS score were analyzed separately, there were no significant differences between the untreated and treated mice fed a WDF, only with the healthy mice (Fig. 6F). The inflammatory infiltrates were also counted in high-magnification fields, with the RM-LNC group again being the only group not significantly different from the healthy mice (Fig. 6G). Representative liver sections stained with H&E were selected and are concordant with the NAS score obtained, with all groups fed a WDF having similar scores (CTRL WDF: x^−^=7.3 ± 0.2; EXE SC: x^−^=6.9 ± 0.4; EXE: x^−^=7.7 ± 0.2; EXE-RM-LNC: x^−^=7.6 ± 0.3) with a slight decrease in the RM-LNC group (RM-LNC: x^−^=6.1 ± 0.4), as seen in the histological images (Fig. 6H).

Further analyses were conducted to assess neutrophil infiltration by performing a LY-6G staining (LY-6G+ area/ total area stained). Neutrophils are implicated in the progression of NAFLD and in amplifying the inflammatory conditions by producing myeloperoxidase (MPO), which leads to increased production of reactive oxygen species (ROS), resulting in cellular damage [20]. Significant differences were observed between the CTRL ND and the EXE group (*P* = 0.023) and with the RM-LNC (*P* = 0.039) group (Supplementary information, Fig. S7A). In the mice fed a ND, neutrophils were present to a lesser extent than in the remaining groups, and their distribution was shown to be more scattered throughout the tissue, while in the groups fed a WDF, the neutrophils tended to be more aggregated (Supplementary information, Fig. S7B).

In this model, there were no significant improvements in the liver when compared to the untreated mice fed a WDF, which could be due in part to the presence of fructose in the drinking water. Fructose can lead to an increase in fat storage in the liver via the stimulation of the de novo lipogenesis pathway [21,22]. It has been shown that the use of fructose in combination with a western diet leads to increased lipid deposition in the liver and to loss of insulin sensitivity while maintaining caloric intake [23]. Chronic consumption of fructose may also lead to increased levels of endotoxins reaching the liver via the portal vein, helping to further increase the inflammation in NAFLD [24]. This increase in inflammation can be seen in the number of inflammatory infiltrates counted in high-magnification fields with a 6- to 8-fold increase in the RM-LNC (x^−^=1.3 ± 0.5 foci vs. x^−^=5.8 ± 1.3 foci) and EXE-RM-LNC (x^−^=1.0 ± 0.4 foci vs. x^−^=8.1 ± 1.6 foci) groups and only a 2- to 3-fold increase in the remaining groups when compared to the *foz/foz* mice (Fig. 5G and 6G).

## 5 Conclusion

Both unloaded and exenatide-loaded lipid nanocapsules exerted a pronounced effect on glucose homeostasis and insulin resistance compared to the other treatment groups, in the two animal models of early NASH studied, demonstrating that the observed effect is not model specific. Moreover, we could halt the progression of the disease and be more efficient at treating the metabolic disorders associated with NAFLD when orally administering exenatide within lipid nanocapsules than subcutaneously. Our therapeutic approach had an impact on the steatosis and inflammation observed in the liver. Lipid nanocapsules alone reduced liver inflammation in both animal models, and it would be worth studying the mechanisms and/or the lipid composition responsible for this effect. This approach has potential for combination therapies via the oral route, potentially leading to novel approaches to NAFLD treatment and oral incretin-based nanomedicine.

## Supplementary Material

Supplementary information

## Figures and Tables

**Table 1:** Composition of EXE-RM-LNCs.

Composition LNCs	
Lipoid® S100 (mg)	13.4
Kolliphor® HS15 (mg)	120
Peceol® (mg)	85.5
Labrafac® WL 1349 (mg)	769.5
NaCl (mg)	50
MilliQ Water (μL)	1025
Cold MilliQ Water (μL)	2500
Composition RMs	
Labrafac® WL 1349 (mg)	500
Span® 80 (mg)	100
Exenatide solution (30 mg/mL) in Water (μL)	50

**Table 2:** Physicochemical properties of exenatide-loaded and unloaded lipid nanocapsules (PDI: polydispersity index; EE: encapsulation efficiency).

	EXE RM LNCs	RM LNCs
Size (nm)	181.7 ± 7.7	184.6 ± 8.8
PDI	0.1 ± 0.05	0.1 ± 0.06
Zeta Potential (mV)	−6.5 ± 1.9	−7.0 ± 2.6
EE (%) (*n* = 3)	78.2 ± 4.2	–

## Data Availability

Data will be made available on request.
