# Supplementary material for: Exploiting the biological effect exerted by lipid nanocapsules in non-alcoholic fatty liver disease
Source: J Control Release. 2023 Apr;356:542–53. doi: 10.1016/j.jconrel.2023.03.012 (PMC7614370; doi:10.1016/j.jconrel.2023.03.012)
Supplement: Supplementary file 1 — Supplementary material [file mmc1.docx]

**Exploiting the Biological Effect Exerted by Lipid Nanocapsules in Non-Alcoholic Fatty Liver Disease**

Inês Domingues^1^, Cecilia Bohns Michalowski^1^, Valentina Marotti^1^, Wunan Zhang^1^, Matthias Van Hul^2,3^, Patrice D. Cani^2,3^, Isabelle Leclercq^4*^, Ana Beloqui^1,3*^

^1^ UCLouvain, Université catholique de Louvain, Louvain Drug Research Institute, Advanced Drug Delivery and Biomaterials Group, Avenue Emmanuel Mounier 73, 1200 Brussels, Belgium

^2^ UCLouvain, Université catholique de Louvain, Louvain Drug Research Institute, Metabolism and Nutrition Group, Avenue Emmanuel Mounier 73, 1200 Brussels, Belgium

^3^ WELBIO (Walloon Excellence in Life sciences and BIOtechnology), WELBIO department, WEL Research Institute, Avenue Pasteur, 6, 1300 Wavre (Belgium)

^4^ UCLouvain, Université catholique de Louvain, Institute of Experimental and Clinical Research, Laboratory of Hepato-Gastroenterology, Avenue Emmanuel Mounier 53, 1200 Brussels, Belgium

*Corresponding authors: [isabelle.leclercq@uclouvain.be](mailto:isabelle.leclercq@uclouvain.be) & [ana.beloqui@uclouvain.be](mailto:ana.beloqui@uclouvain.be)

**Supplementary Figures**


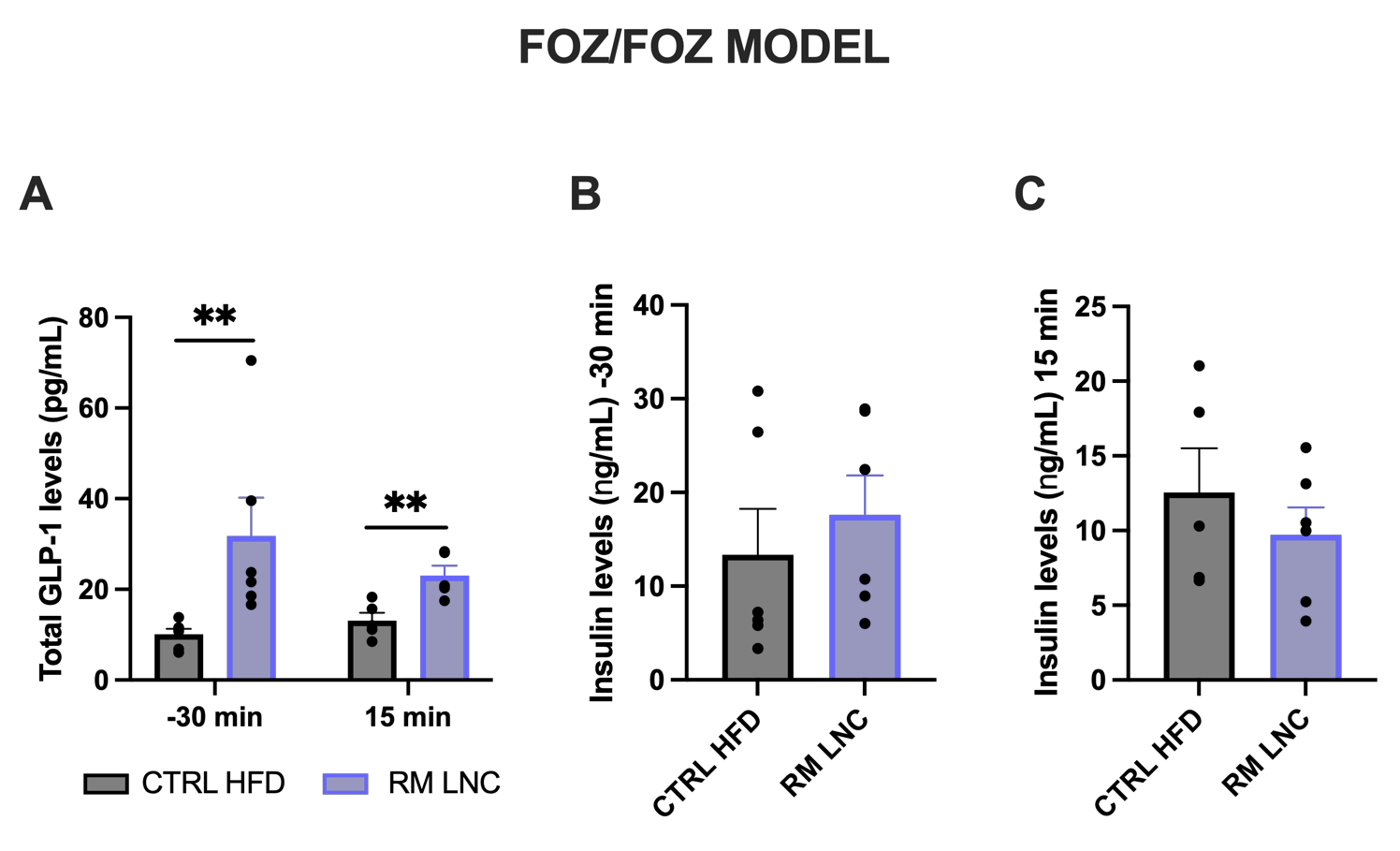


**Figure S1:** RM-LNC impact on GLP-1 secretion in NAFLD. (A) Plasmatic total GLP-1 levels (pg/mL), (B, C) Plasmatic insulin levels (ng/mL) measured 30 min before and 15 min after glucose challenge. Data represented as mean±SEM (n=5-6). *P* values in (A) were determined by unpaired t-test or Mann-Whitney test (***P*<0.01).


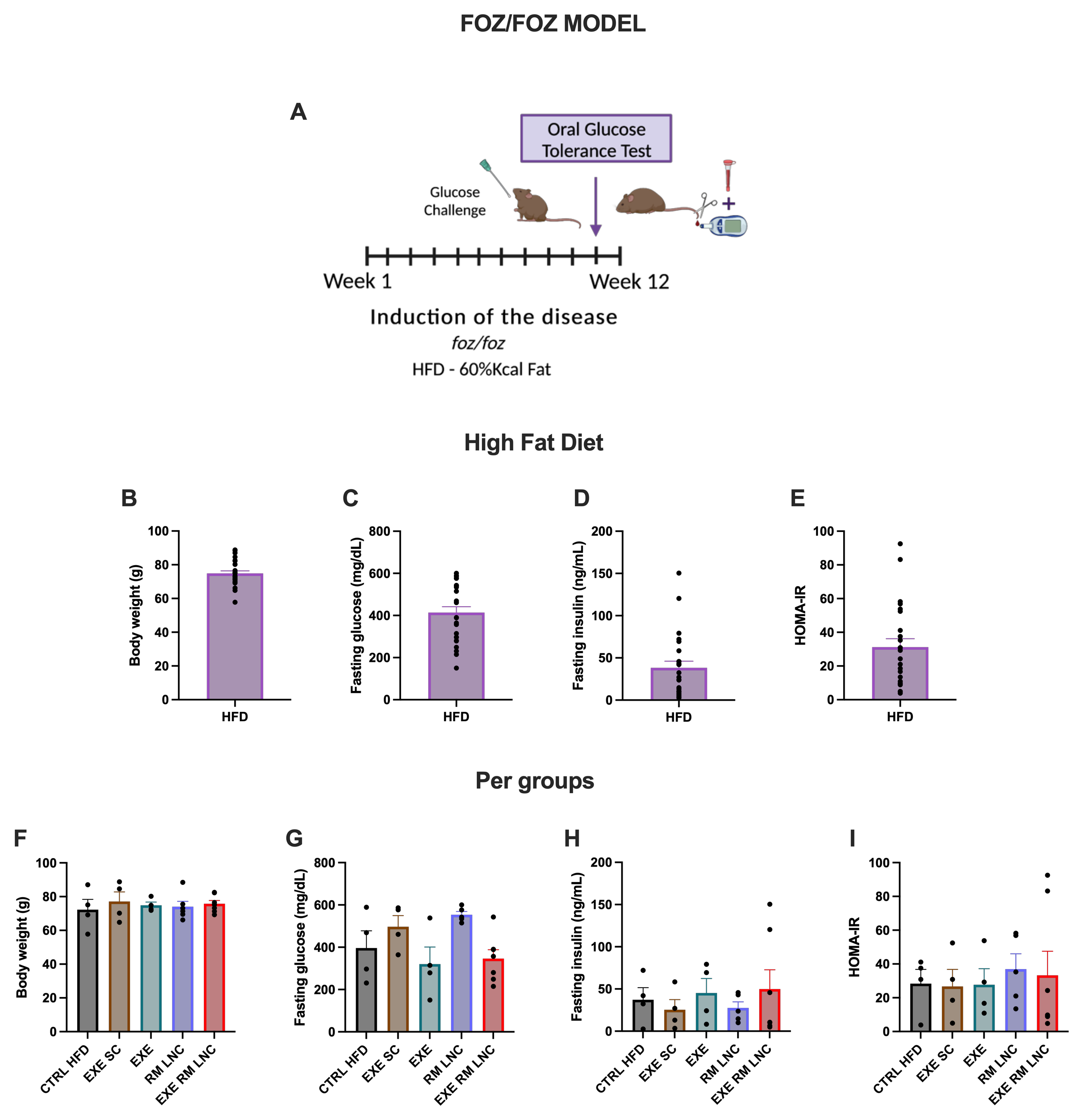


**Figure S2:** Genetic model of NASH – *foz/foz* model: disease induction. (A) Schematic representation of the disease induction period before starting the treatment (12 weeks), (B; F) Body weight (g), (C; G) Fasting glucose (mg/dL), (D; H) Fasting insulin (ng/mL), (E; I) Homeostatic Model Assessment of Insulin Resistance (HOMA-IR) calculated using the equation [fasting glucose (mg/dL) x fasting insulin (ng/mL)/405] at week 11. Results (F-I): the mice were randomized to obtain body weight matched groups. Data represented as mean±SEM (n=25) or (n=4-6).


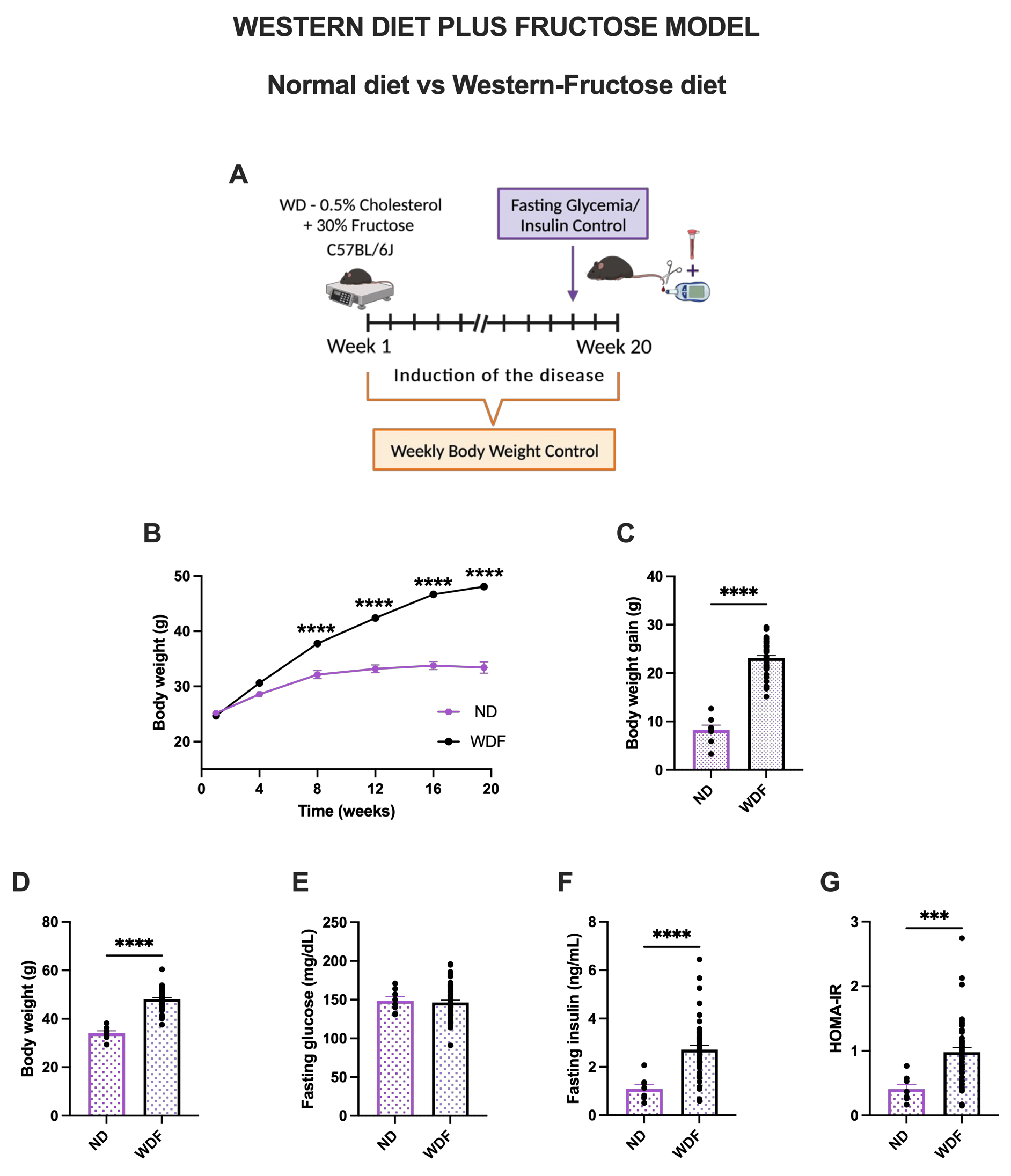


**Figure S3:** Dietary model of NASH – Western diet plus fructose model: disease induction. (A) Schematic representation of the disease induction period before starting the treatment (20 weeks), (B) Body weight (g) throughout the 20 weeks, (C) Body weight gain (g), (D) Body weight (g), (E) Fasting glucose (mg/dL), (F) Fasting insulin (ng/mL), (G) Homeostatic Model Assessment of Insulin Resistance (HOMA-IR) calculated using the equation [fasting glucose (mg/dL) x fasting insulin (ng/mL)/405]. Data shown in (D-G) were obtained at week 18 of disease induction. Data represented as mean±SEM (n=10-50). *P* values in (B) were determined by two-way ANOVA followed by Šidák’s post hoc test. *P* values in (C-G) were determined by unpaired t-test or by Mann-Whitney test (****P*<0.001; *****P*<0.0001).

**
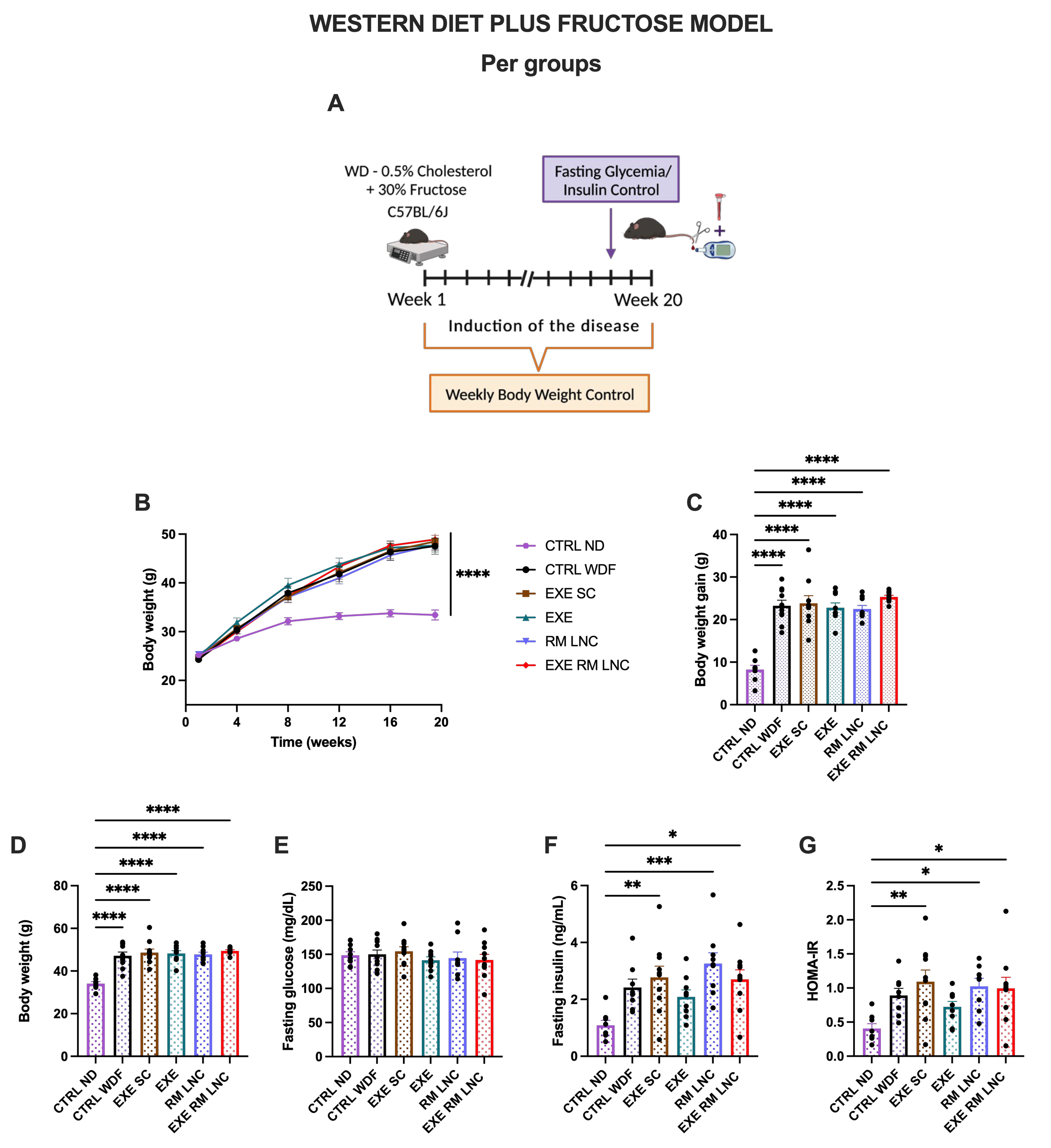
Figure S4:** Dietary model of NASH – Western diet plus fructose model: disease induction (A) Schematic representation of the disease induction period before starting the treatment (20 weeks), (B) Body weight (g) throughout the 20 weeks, (C) Body weight gain (g), (D) Body weight (g), (E) Fasting glucose (mg/dL), (F) Fasting insulin (ng/mL), (G) Homeostatic Model Assessment of Insulin Resistance (HOMA-IR) calculated using the equation [fasting glucose (mg/dL) x fasting insulin (ng/mL)/405]. Data shown in (D-G) were obtained at week 18 of disease induction. Data represented as mean±SEM (n=8-10). *P* values in (B) were determined by a two-way ANOVA flowed by Tukey’s post hoc test (CTRL ND vs EXE SC/EXE/LNC/EXE LNC). *P* values in (C-G) were determined by ordinary one-way ANOVA followed by Tukey’s post hoc test or by Kruskal-Wallis followed by Dunn’s post hoc test (**P*<0.05; ***P*<0.01; ****P*<0.001; *****P*<0.0001).


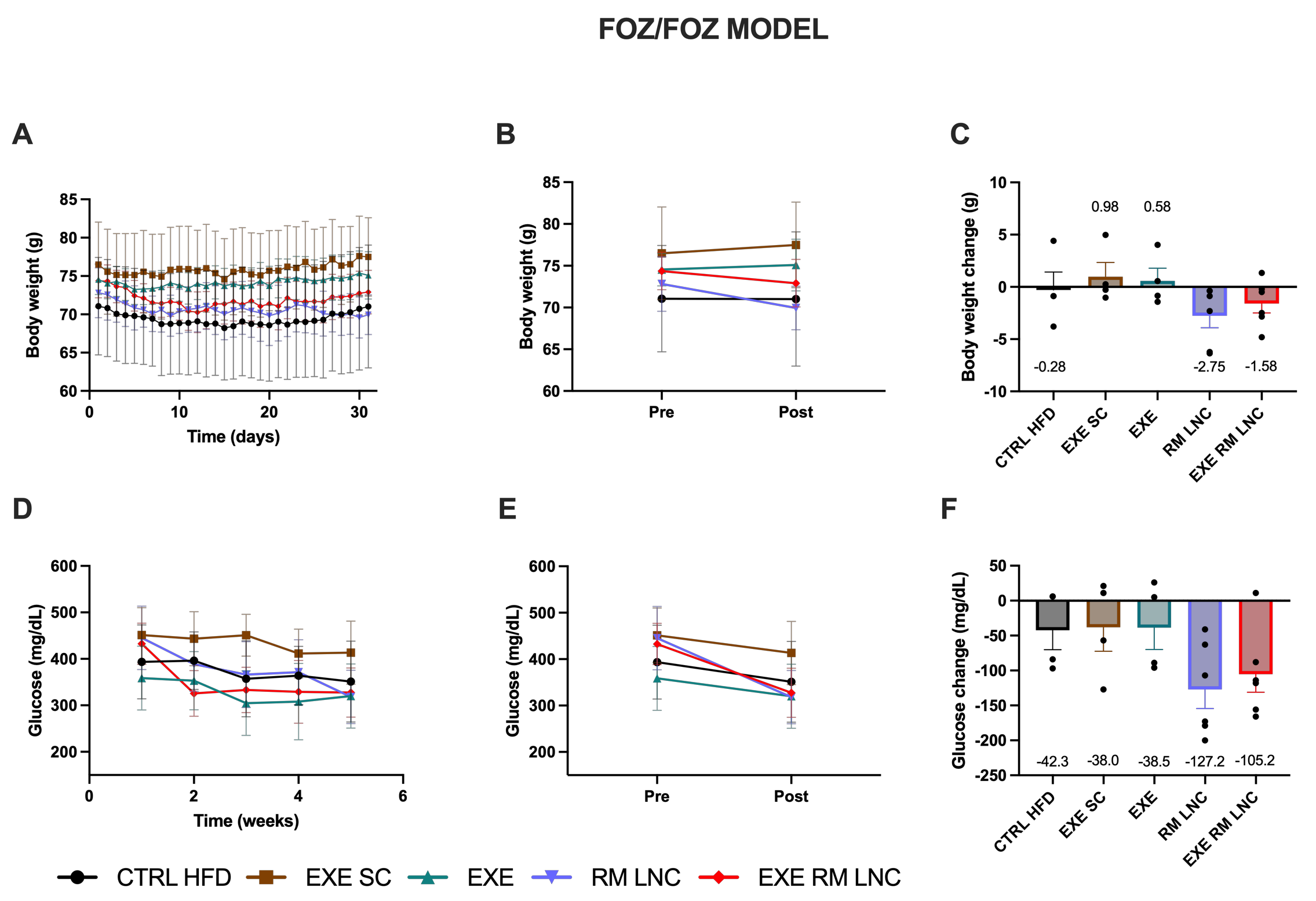


**Figure S5:** EXE-RM-LNC have an impact on glucose homeostasis and insulin resistance in the *foz/foz* early NASH model. (A) Body weight (g), (B) Pre/Post: Body weight (g), (C) Body weight change (g), (D) Non-fasting glucose (mg/dL), (E) Pre/Post: Non-fasting glucose (mg/dL), (F) Non-fasting glucose change (mg/dL) Pre: beginning of treatment; Post: end of treatment. Results in (C; F) were calculated by subtracting the post values from the pre values. Data represented as mean±SEM (n=4-6).


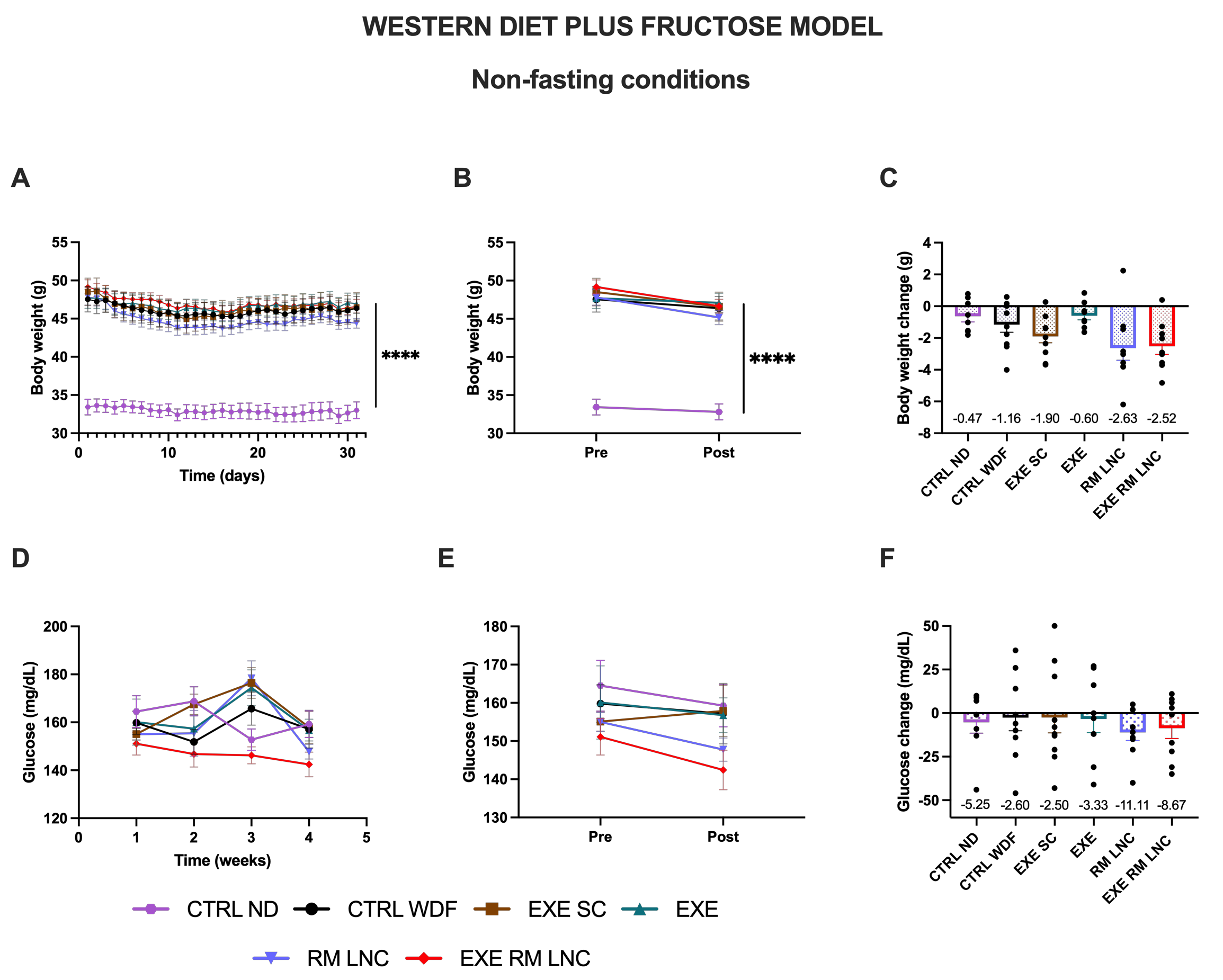


**Figure S6:** EXE-RM-LNC have an impact on glucose homeostasis and insulin resistance in the WDF model of early NASH. (A) Body weight (g), (B) Pre/Post: Body weight (g), (C) Body weight change (g), (D) Non-fasting glucose (mg/dL), (E) Pre/Post: Non-fasting glucose (mg/dL), (F) Non-fasting glucose change (mg/dL). Pre: beginning of treatment; Post: end of treatment. Results in (C; F) were calculated by subtracting the post values from the pre values. P values in (A; B) were determined by a two-way ANOVA followed by Tukey’s post hoc test (CTRL ND vs EXE SC/EXE/LNC/EXE LNC) (*****P*<0.0001). Data represented as mean±SEM (n=8-10).


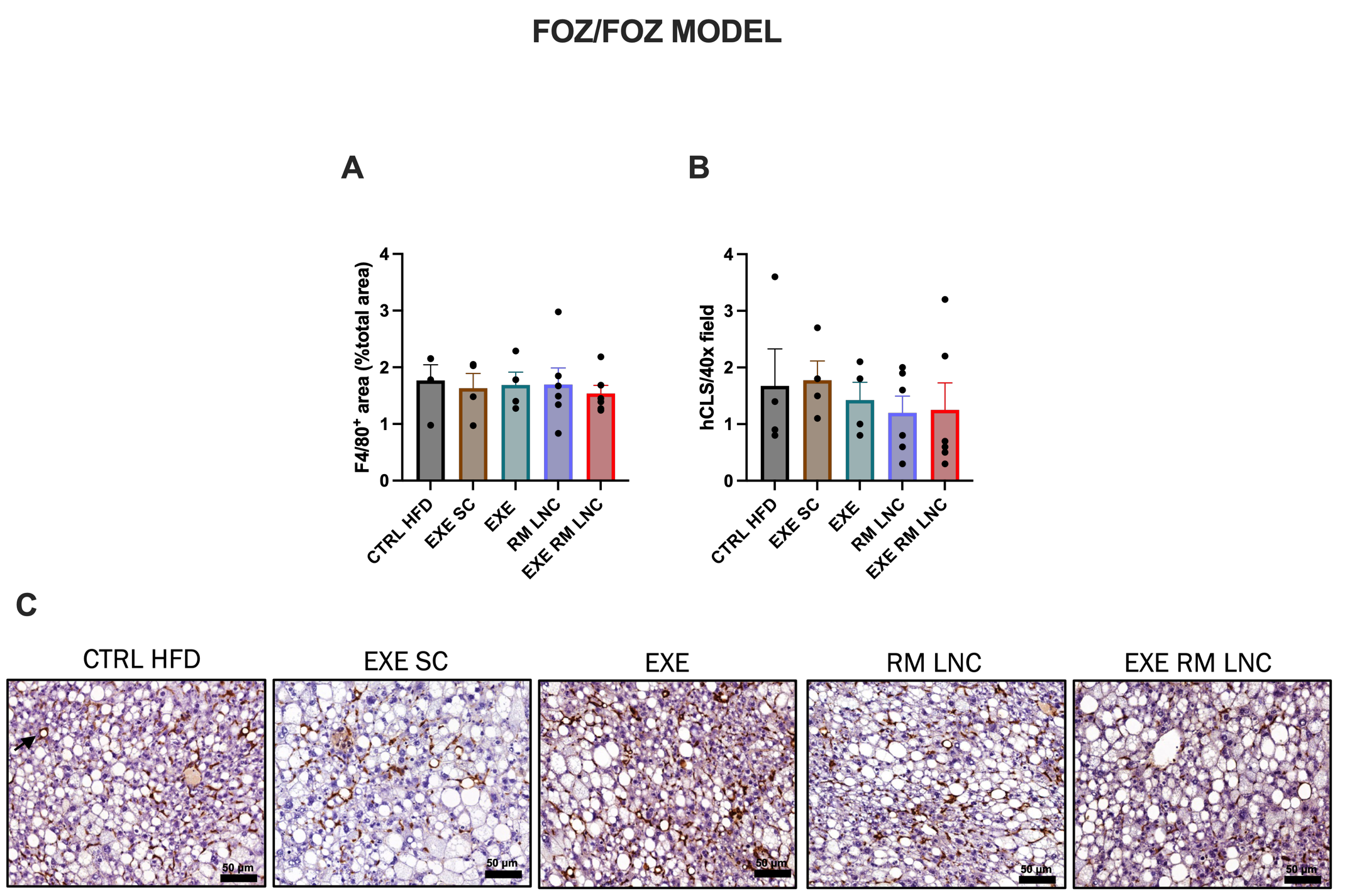


**Figure S7:** EXE-RM-LNC impact on the inflammation in the *foz/foz* early NASH model (A) Quantification of macrophages in liver sections, (B) Quantification of hepatic crown-like structures in the liver (10 fields per mouse), (C) Representative F4/80 staining of liver sections with hepatic crown-like structures (black arrow) (scale bar: 50 μm). Data represented as mean±SEM (n=4-6).

9


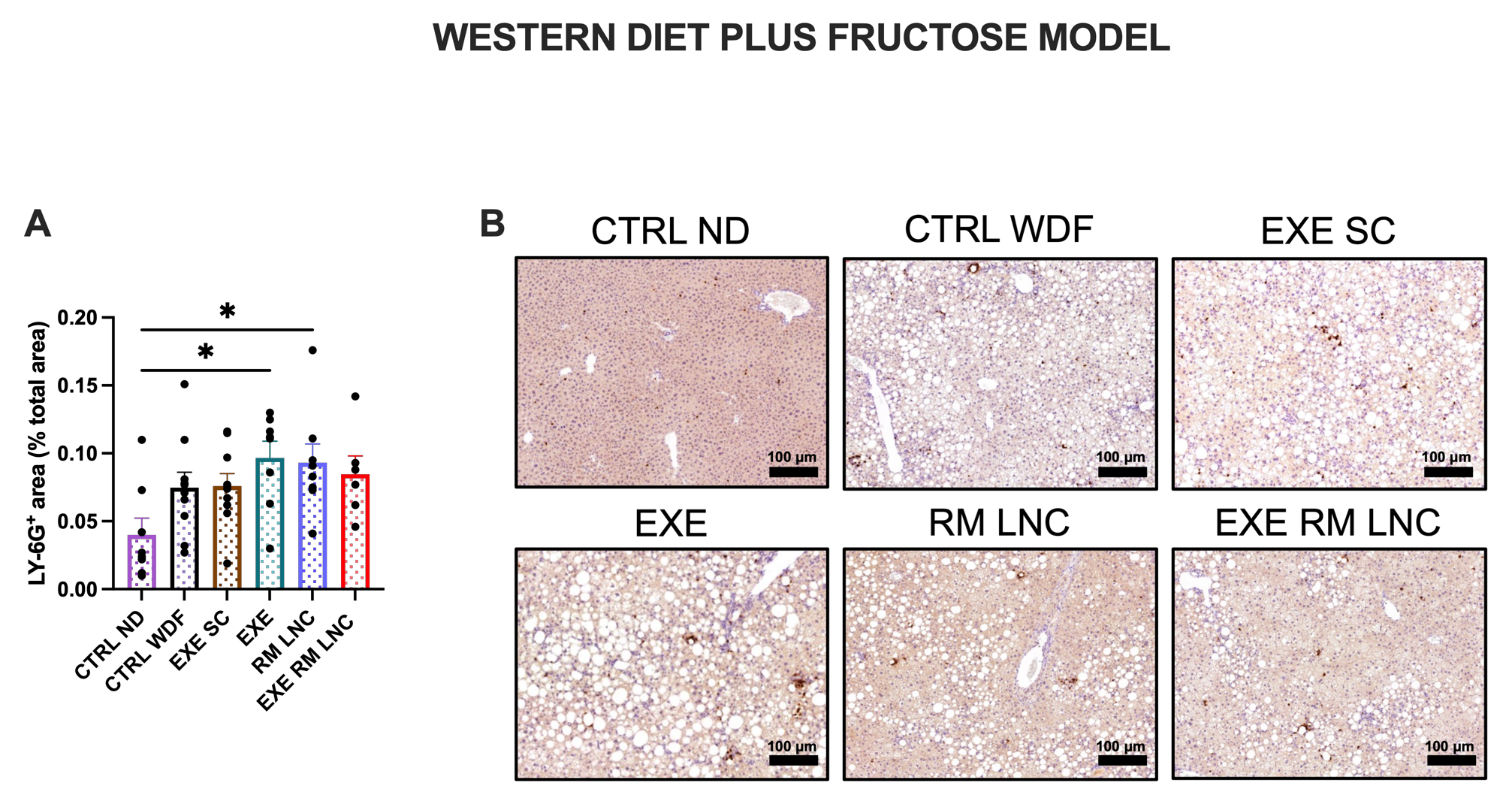


**Figure S8:** EXE-RM-LNC impact on inflammation in a WDF model of early NASH (A) Quantification of neutrophils in liver sections, (B) Representative LY-6G staining of liver sections (scale bar: 100 μm). Data represented as mean±SEM (n=8-10). *P* values in (A) were determined by ordinary one-way ANOVA followed by Tukey’s post hoc test (**P*<0.05).
